# Regulation of Ant Foraging: A Review of the Role of Information Use and Personality

**DOI:** 10.3389/fpsyg.2020.00734

**Published:** 2020-04-28

**Authors:** Swetashree Kolay, Raphaël Boulay, Patrizia d’Ettorre

**Affiliations:** ^1^Laboratory of Experimental and Comparative Ethology (LEEC) UR4443, Université Sorbonne Paris Nord, Villetaneuse, France; ^2^Institute of Insect Biology (IRBI), UMR CNRS 7261, University of Tours, Tours, France; ^3^Institut Universitaire de France (IUF), Paris, France

**Keywords:** behavioral plasticity, collective behavior, foraging, recruitment, social insects

## Abstract

Animals live in heterogeneous environments where food resources are transient and have to be exploited rapidly. Ants show a wide range of foraging strategies and this activity is tightly regulated irrespective of the mode of recruitment used. Individual foragers base their decision to forage on information received from nestmates (social information). Transmission of information can be in the form of direct physical interactions such as antennation or indirect exchange of information such as laying of pheromone trails. Foragers also rely on information from their internal states or experience (personal information). The interaction between these two sources of information gives rise to plasticity in foraging behavior. Recent studies have examined the role of personality (consistent inter-individual variation in behavioral traits) during ant foraging. Since colonies differ from each other in the distribution of personalities of their members, colonies may consistently differ in behavioral traits, giving rise to colony level personality. However, the interaction between information use and personality, especially at the individual level, remains unexplored. Here, we briefly summarize the literature on the effect of social and personal information on the regulation of ant foraging and the effect of personality on this behavior. We point out that a more focused examination of the interplay between personality and information use will help us understand how behavioral plasticity in the context of foraging is shaped at the colony and individual levels.

## Introduction

Animals live in complex and heterogeneous environments with fluctuating resource availability. Effective decision making in different contexts is critical to their survival and fitness. In group-living species, including humans, collective decisions emerge from the actions of individual group members. The behavior of each individual is modulated by the behavior of others and affects the group as a whole (Conradt and List, 2009). Social insects such as ants live in colonies which consist of tens to millions of individuals and function as self-organized systems without central leadership (Jandt and Gordon, 2016). This is possible due to exchange of information among nest mates which allows individuals to coordinate their activities, thus maximizing colony efficiency (Duarte et al., 2011). Here, we first describe the different modes of foraging recruitment in ants. We then discuss how the use of different sources of information and consistent variation in behavioral traits among colonies and individuals contribute to bring about context-dependent plasticity in ant foraging behavior.

## Recruitment Mechanisms in Ants

Of the more than 16,000 recorded species of ants (Bolton, 2020), foraging recruitment has been studied in detail in only a handful of species and they use different strategies to recruit nestmates to the food source (reviewed in Holldobler and Wilson, 1990).

(1) Tandem running: In this mode of recruitment, a recruiter who knows the location of the food source leads one nestmate at a time from the nest to the food source (reviewed in Franklin, 2014). Cohesion between the tandem pair is maintained by contiguous physical contact between the two ants and/or by short-range chemical signals emitted by the recruiter. The number of recruited foragers is largely proportional to the number of successful scouts as the recruits have to be actively guided by the recruiters on each trip.

(2) Mass recruitment using chemical trails: Here, ants that find a food source lay a pheromone trail while returning to the nest and this triggers the recruitment of nestmates (Wilson, 1962). The recruits, in turn, reinforce the trail while returning to the nest which leads to further recruitment (reviewed in Czaczkes et al., 2015). The number of ants joining the trail is a function of its strength. To prevent excessive mobilization of foragers, reinforcement of the trail is downregulated or an inhibitory signal may be produced.

(3) Group recruitment: In this case, one ant summons a few nestmates at a time from the nest and the recruited ants follow the leader ant closely to reach the food source. Although a trail may be laid by the leader, it is not enough to stimulate recruitment alone. Here, as in tandem running, the number of recruits will be determined by the number of successful scouts (Holldobler and Wilson, 1990).

(4) Group retrieving based on distant homing: This is another mode of recruitment that has been proposed where individual scouts appear to transmit information about a distant food source to groups of recruits through direct physical contact such as antennation. No other cues such as chemical trails or direct guiding is required for recruitment (Reznikova, 2008).

Independent of the recruitment strategy, the recruit receives information about the food source such as scent and type of food from the recruiter. This information helps the recruit in making its foraging decisions such as whether to initiate foraging, which food source to select and which path to follow.

## Use of Social and Personal Information in Regulation of Foraging

In social insect colonies, particularly in large ones, group members only have access to local information based on their position in the nest and the nestmates present in the vicinity (Mersch, 2016). Thus, foragers may not have direct access to information about food requirement of the colony. In addition, foragers have to choose from a range of alternatives such as type and location of food and path to the food source. Effective communication among workers is critical in regulating foraging activity. Individual foragers within a colony base their decisions to engage in foraging on social information received from their nestmates and their personal information. Social information may be obtained via direct interactions with nestmates and/or by indirect exchange of information. Foragers also rely on personal information based on their internal states, their interactions with the environment or their past experiences (reviewed in Dall et al., 2005). The majority of studies on information use during foraging have focused on trail laying species and there is little information available on species using other recruitment strategies.

### Social Information

In trail laying species, the chemical trail, which usually contains multiple pheromones, transmits information about the food source to potential recruits. The number of ants laying trail pheromones as well as the intensity of pheromone deposition is related to the quality of food in several species such as the black garden ant *Lasius niger* (Mailleux et al., 2003; Detrain and Prieur, 2014), the pavement ant *Tetramorium caespitum* (Collignon and Detrain, 2010) and the Pharaoh’s ant *Monomorium pharaonis* (Jackson and Châline, 2007). However, it has recently been suggested that pheromone trails may actually provide rather inaccurate information about food quality (Czaczkes et al., 2019). In addition to recruiting workers from the nest, the trail also stimulates scouts who are already outside to join the trail, as has been seen in the neotropical species *Pheidole oxyops* (Czaczkes and Ratnieks, 2012). Use of a combination of two pheromones – a long-lasting pheromone and a shorter lasting one – which allows colonies to track foraging resources more effectively while maintaining foraging cohesion has been documented in *M. pharaonis* (Jackson et al., 2006), the army ant *Leptogenys distinguenda* (Witte and Maschwitz, 2002) and the big headed ant *Pheidole megacephala* (Dussutour et al., 2009). In order to downregulate recruitment to a food source, *L. niger* foragers reduce pheromone deposition on trails that have already been heavily marked by trail pheromones (Czaczkes et al., 2013a) while a no-entry pheromone appears to repel foragers from unrewarding paths in *M. pharaonis* (Robinson et al., 2005).

Much information can be exchanged through direct physical contact between nestmates. High collision rates between foragers on a trail cause them to reduce pheromone deposition (Czaczkes et al., 2013b) or drive some ants to choose an alternate path in *L. niger* (Dussutour et al., 2004). Encounters between returning and outgoing foragers convey information about the partner’s identity, the type of food being exploited and the richness of the food source. Leaf-cutter ants, *Atta cephalotes*, which collect leaves for the symbiotic fungus gardens inside their nests, use encounters on the trail to exchange information about the type of leaves being collected (Farji-Brener et al., 2010). Contact with food residues on a recruiter’s body informs the recruits about the food type that is being exploited and this increases the success of finding the food patch in *L. niger* (Le Breton and Fourcassié, 2004). In tandem running species, continuous antennal contact between the recruiter and the recruit is essential for progression of the tandem run (Richardson et al., 2007). During each tandem run, the recruits get the opportunity to learn the path to the food source and they, in turn, recruit other nestmates (Franklin and Franks, 2012). Scouts of *Formica polyctena* appear to convey quantitative information about the location of food sources to recruits through antennal contact (Reznikova and Ryabko, 2011).

Cuticular hydrocarbons (CHCs) comprising of a blend of different hydrocarbons are present in a wax layer on the insect body (Blomquist and Bagneres, 2010). The CHC profile of individuals is related to their task repertoire and can inform the task decisions of nestmates. For example, it has been shown in the red harvester ant *Pogonomyrmex barbatus* that foragers have a higher ratio of saturated, linear hydrocarbons to linear alkenes and branched alkanes on the cuticle as compared to workers performing tasks inside the nest (Wagner et al., 2001). This forager-specific CHC profile not only helps in preventing water loss, which is critical as these ants forage in hot and dry conditions, but has also a communicative function by affecting task decisions of others (Greene and Gordon, 2003). Brief antennal contacts with a returning forager at the nest entrance allows inactive foragers to assess its CHC profile and whether it is carrying food. The combination of both odors is required to stimulate foraging in this species (Greene et al., 2013).

### Personal Information

Personal information may be related to an individual’s physiology with leaner individuals making extra foraging trips in response to an increased demand for foraging, as has been observed in *Temnothorax albipennis* (Robinson et al., 2009a). A forager’s decision to initiate recruitment may be based on an internal response threshold such as ingestion of a desired volume of liquid food at a food source as shown in *L. niger* (Mailleux et al., 2000) and this threshold increases under conditions of starvation (Mailleux et al., 2006). Enhanced response to recruitment signals after a period of starvation has been observed in species such as *L. niger* (Mailleux et al., 2011), *Linepithema humile* and *Euprenolepis procera* (von Thienen et al., 2016). Personal information may also be based on prior experience. In *Ooceraea biroi*, foraging tendency among individuals of the same age is strongly correlated to successful foraging experiences in the past (Ravary et al., 2007). In two *Formica* species, individual foragers tend to return to sites where they have had positive experiences in the past (Tanner, 2009). Tandem running recruiters use visual landmarks to improve upon previously learnt routes (Pratt et al., 2001) and likelihood of becoming a recruiter increases with experience (Franklin et al., 2012).

### Interplay Between Social and Personal Information

Individual ants extensively use both social and personal information to make foraging decisions but reliance on a particular source of information depends on its content relative to other sources. *L. niger* uses a combination of route memory and trail pheromones to maximize foraging efficiency (Czaczkes et al., 2011). In species that use visual cues to form route memory, low light conditions may lead to reliance on social signals rather than reliance on personal memories as has been reported in *L. niger* (Jones et al., 2019) and *Formica pratensis* (Beugnon and Fourcassié, 1988). In *T. albipennis*, contact with returning foragers at the nest entrance causes bouts of activity. In the absence of this social information, physiology of individual foragers predicts which ants will leave the nest as mentioned earlier (Robinson et al., 2009b). When there is a conflict between social and private information, individuals depend on personal information to make foraging decisions in many species such as *Acromyrmex subterraneus* (Almeida et al., 2018), *Formica lugubris* (Fourcassié and Beugnon, 1988), *L. niger* (Aron et al., 1993; Grüter et al., 2011), and *Paraponera clavata* (Harrison et al., 1989). The reverse, i.e., preference for social information over private information, has been observed in *L. humile* (Aron et al., 1993), *Atta cephalotes*, *Atta laevigata*, and *Acromyrmex octospinosus* (Vilela et al., 1987) while no clear preference for either is shown in *Iridomyrmex purpureus* (Middleton et al., 2018). It has been suggested that ants prioritize social or personal information based on the information content of each source and choose the source that provides more detailed, accurate and reliable information about the food source. Thus, a change in the accuracy and reliability of information from one of the sources may cause individuals to switch their choice of information source as has been demonstrated in *L. niger* (Czaczkes et al., 2019).

## Role of Individual and Collective Personality

The field of animal personality – defined as consistent inter-individual differences in behavioral traits across time and/or context – has seen rapid progress in the last two decades and personality traits have been documented in a wide range of taxa (Dingemanse et al., 2010). In social insects, in addition to individual differences in personality traits, groups differ consistently from each other in task performance and regulation of activity, giving rise to group level personality (Webster and Ward, 2011). For example, colonies may vary consistently in the baseline number of foragers that leave the nest to collect food. Group personality, or the particular configuration of behaviors expressed by the group, is likely to emerge from the differential aggregation of individual personalities comprising the colony or by external factors that vary consistently among colonies and affect colony behavior (Pinter-Wollman, 2012). Since the colony is the reproductive unit (Bourke, 2011), consistent behavioral variation among colonies may lead to fitness differences among them (Gordon, 2013). Certain behaviors such as boldness and aggression may be correlated at the population level and such suites of correlated behaviors are defined as behavioral syndromes (Sih et al., 2012). Within the behavioral syndrome expressed at the population level, each individual has a behavioral type; for instance, some individuals may be more bold and aggressive than others (Bell, 2007). Different behavioral types can coexist within a population (Wolf and Weissing, 2010).

### Collective Personality

Several studies have looked at variations in behavioral traits at the level of the colony in different species of ants. In *P. barbatus*, colonies exhibit variation not only in the rate at which scouts leave the nest to search for food but also the ratio of outgoing foragers to returning foragers (Gordon et al., 2011). These differences in foraging activity among colonies persist from year to year (Gordon et al., 2013). Colonies of *Pogonomyrmex occidentalis* show consistent variation in the temporal pattern of foraging activity and also in the thermal range across which they forage (Cole et al., 2010). Colonies of *Temnothorax rugatulus* show consistency in their foraging effort and how they respond to different types of resources (Bengston and Dornhaus, 2014). Colonies of *Aphaenogaster senilis* that are more aggressive, readily explore novel environments and forage at higher temperatures (proactive colonies) are more successful than reactive colonies in retrieving food during intraspecific competition but suffer higher mortality rates (Blight et al., 2016). Colonies of *S. invicta* show persistent variation in foraging behaviors which is significantly related to colony growth (Bockoven et al., 2015). In *L. niger*, exploratory activity varies consistently among colonies and colonies with higher levels of exploratory activity discover and exploit food sources faster (Pasquier and Grüter, 2016). Colony personality is influenced by nest structure in *Messor andrei* and the foraging activity of colonies is consistent as long as they occupy the same nest sites (Pinter-Wollman et al., 2012). Behavioral syndromes have been identified at multiple levels in *Myrmica* ants where boldness is correlated with aggression at the caste level and with sociability at the colony level (Chapman et al., 2011).

### Individual Personality

At the individual level, consistent variations in forager behavior has been observed in several species. In scouts of *L. niger*, intake of a desired volume of liquid is key to initiation of recruitment. This desired volume is specific to each individual, irrespective of its size, and remains constant over successive trips to a food source. There is also inter-individual difference in the persistence of trail-laying with some foragers never laying trails (Mailleux et al., 2005). Caste-based differences in personality traits have been identified in a few species. In *Myrmica rubra*, foragers are more active, exploratory, aggressive and attracted to light than workers who worked inside the nest (Pamminger et al., 2014). Foragers of *Camponotus aethiops* show better learning abilities and higher sucrose responsiveness than the nurses (Perez et al., 2013). However, it is not clear from these studies whether personality is related to the age of workers which determines which tasks they will perform. A few empirical studies have explicitly investigated the influence of personality of individual foragers on their foraging behavior. Learning performance was found to be correlated to exploration behavior in *C. aethiops* foragers with active explorers being slower to learn a task than less active ones (Udino et al., 2017).

## Future Directions

Ant foraging behavior has been the focus of intensive studies for decades, yet much remains to be understood. We highlight a couple of avenues for further research that will give us a more comprehensive understanding of how individual and group level personality may affect the regulation of foraging in ants.

**FIGURE 1:**
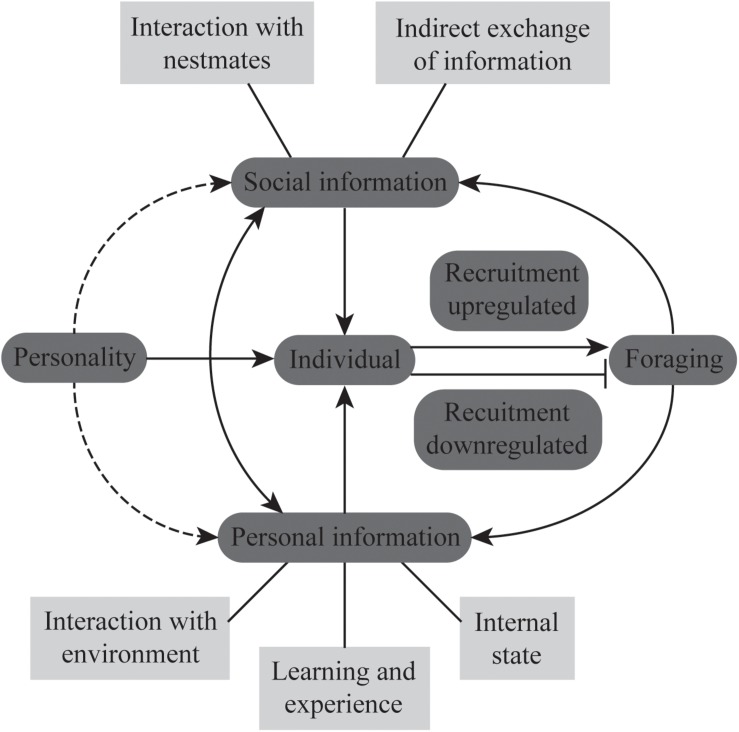
The schematic represents the interplay between social and personal information and personality in regulating foraging behavior of individuals in ant colonies. The solid lines indicate known paths of interactions and the dotted lines indicate expected paths of interactions as discussed in the text. The arrowheads indicate the direction of the interaction. The different sources of information (social and personal) have been listed in the light gray boxes. The foraging decisions of individuals will determine collective foraging at the level of the colony.

(1) Numerous studies have separately investigated how the use of different sources of information and personality, largely at the colony level, influence foraging. However, the interaction between these two factors, particularly at the level of individual foragers, in giving rise to plasticity in foraging behavior (as shown in Figure 1) remains relatively unexplored. We predict that individuals with different personalities will vary in the manner in which they perceive and use information, prioritize personal and social information, and in their learning abilities (Carere and Locurto, 2011; Sih and del Giudice, 2012). As a result, they will differ in task specializations. For example, individuals who are bolder and show more exploratory activity may more readily become scouts who go out in the initial search for food. These individuals should also have more flexible learning abilities and rely more on personal information. Foragers who vary in their foraging strategies as in *Ectatomma ruidum* (McGlynn et al., 2015) or in their resource specialization as in *Formica aquilonia* (Iakovlev and Reznikova, 2019) should also vary in their personality traits and cognitive abilities. Since the task repertoire of individual ants changes with age, further studies are also required to understand whether personality traits of individuals remain constant across their lifetimes and how this affects their task choice and task performance at different stages of their life.

(2) In social insects, colony personality is determined by the distribution of individual personalities within the group and differences in the underlying personality distributions will affect collective behavior. Most studies on foraging regulation have been done at the colony level by essentially looking at the average behavior of the group as a whole. Variations in behavioral traits at the individual level are not adequately captured by such studies (for example, Pamir et al., 2011 in honeybees). Thus, exploration of the distribution of individual personality traits within colonies will shed further light on how collective foraging behavior is shaped. For example, a colony with a higher proportion of individuals who are bold and show high exploratory activity should be able to track changing food resources or detect new food sources more efficiently. Such studies can be done by manipulating group compositions as has been done with ants in other contexts (Carere et al., 2018; Neumann and Pinter-Wollman, 2019).

An integrated analysis of personality and information use at the individual and colony levels will give us a more comprehensive understanding of the emergence and maintenance of context-dependent plasticity in ant foraging behavior.

## Author Contributions

All authors have made substantial intellectual contribution to the work. SK wrote the manuscript with the help of Pd’E.

## Conflict of Interest

The authors declare that the research was conducted in the absence of any commercial or financial relationships that could be construed as a potential conflict of interest.
